# Genome-wide identification and comparative analysis of the *Amino Acid Transporter* (*AAT*) gene family and their roles during *Phaseolus vulgaris* symbioses

**DOI:** 10.1007/s10142-024-01331-0

**Published:** 2024-03-02

**Authors:** Kalpana Nanjareddy, María Fernanda Guerrero-Carrillo, Miguel Lara, Manoj-Kumar Arthikala

**Affiliations:** 1 Ciencias Agrogenómicas, Escuela Nacional de Estudios Superiores Unidad León-Universidad Nacional Autónoma de México (UNAM), Leon, Guanajuato, C.P. 37689 México; 2 Departamento de Biología Molecular de Plantas, Instituto de Biotecnología, Universidad Nacional Autónoma de México (UNAM), Cuernavaca, 62210 Morelos México

**Keywords:** Amino acid transporters, Genome-wide analysis, Legumes, Mycorrhiza, *Phaseolus vulgaris*, *Rhizobium*, Symbiosis

## Abstract

Amino acid transporters (AATs) are essential integral membrane proteins that serve multiple roles, such as facilitating the transport of amino acids across cell membranes. They play a crucial role in the growth and development of plants. *Phaseolus vulgaris*, a significant legume crop, serves as a valuable model for studying root symbiosis. In this study, we have conducted an exploration of the *AAT* gene family in *P. vulgaris*. In this research, we identified 84 *AAT* genes within the *P. vulgaris* genome sequence and categorized them into 12 subfamilies based on their similarity and phylogenetic relationships with *AAT*s found in *Arabidopsis* and rice. Interestingly, these *AAT* genes were not evenly distributed across the chromosomes of *P. vulgaris* . Instead, there was an unusual concentration of these genes located toward the outer edges of chromosomal arms. Upon conducting motif analysis and gene structural analysis, we observed a consistent presence of similar motifs and an intron-exon distribution pattern among the subfamilies. When we analyzed the expression profiles of *PvAAT* genes, we noted tissue-specific expression patterns. Furthermore, our investigation into *AAT* gene expression under rhizobial and mycorrhizal symbiotic conditions revealed that certain genes exhibited high levels of expression. Specifically, *ATLa5* and *LHT2* was notably upregulated under both symbiotic conditions. These findings point towards a potential role of AATs in the context of rhizobial and mycorrhizal symbiosis in *P. vulgaris*, in addition to their well-established regulatory functions.

**Supplementary Information:**

The online version contains supplementary material available at 10.1007/s10142-024-01331-0.

## Introduction

Transporters are a class of membrane proteins that transport nutrients, hormones, and metabolites for the purposes of growth, development, and adaptation to stresses across membranes (Bo et al. 2017). Transmembrane transporters facilitate substrate transport across biological membranes through their transmembrane segments (TMS), which are also known as transmembrane domains (TMDs). Nitrogen is one of the most important nutrients for plant growth and is a major component of many cellular compounds. Plants absorb organic and inorganic nitrogen from the rhizosphere through the root system (Smith and Chalk 2021). In soil, inorganic nitrogen is usually found in the forms of nitrate and ammonium, while organic nitrogen usually exists in the forms of free amino acids, urea, and short peptides (Shen et al. 2003; Kojima et al. 2007; Tegeder and Rentsch 2010; Kotur et al. 2013; Forde 2014).

Plants acquire inorganic nitrogen, rapidly incorporating it into amino acids like glutamine or glutamic acid. Amino acid synthesis occurs in plastids of mesophyll cells and are transported through xylem sap and phloem to support the protein biosynthesis, growth and reproduction (Rentsch et al. 2007; Masclaux-Daubresse et al. 2010; Pratelli and Pilot 2014). Amino acid transporters play a crucial role in regulating processes like leaf senescence, seed germination, and stress response (Frommer et al. 1993; Tegeder and Masclaux-Daubresse 2018).

The uptake and transport of nitrogen-containing molecules by plants is facilitated by an array of amino acid transporters, which have also been known to play a major role in distributing nitrogen throughout the whole plant (Tegeder 2012; Dinkeloo et al. 2018). Most of the amino acid transporters described in plants are proton–amino acid symporters, which is an active transport (Bush 1993). The majority of active transport is driven by proton gradient across the cellular/subcellular membrane. Hence, pH changes in subcellular compartments could regulate the transport activities and determine the direction of transport. The pH change also implies a change in electrical potential difference across the biological membrane. The amino acid uptake is coupled to the proton electrochemical potential difference that is maintained through p-type proton-pumping ATPase (Sze et al. 1999). Taken together these factors influence the transport efficiency and, thus, bring forth the physiological regulations.

Amino acid transporters (AATs) are studied extensively in yeast, mammals and plants are found to be encoded by multiple gene families that encode different classes of amino acid transporters. In plants AATs include two main families, classified based on sequence similarity and uptake properties. The amino acid/auxin permease (AAAP) family, also called the amino acid transporter (ATF) family, and the Amino Acid-Polyamine-Organocation (APC) family (Fischer et al. 1998; Okumoto and Pilot 2011). AAAP family includes 6 subfamilies, amino acid permeases (AAPs), lysine and histidine transporters (LHTs), proline transporters (ProTs), γ-aminobutyric acid transporters (GATs), auxin transporters (AUXs), and aromatic and neutral amino acid transporters (ANTs) (Ortiz-Lopez et al. 2000; Saier et al. 2009). APC subfamily is grouped into three subfamilies cationic amino acid transporters (CATs), amino acid/choline transporters (ACTs) and polyamine H^+^-symporters (PHSs).

The *AAT* genes are identified by the characteristic domains PF01490 (Aa_trans) and PF00324 (Aa_permease). In the AAT superfamily, *Arabidopsis* is found to have 8 genes in Amino acid permease (AAP) subfamily and 6 of them have been functionally characterized. *AtAAP1-8* have been found to be predominately transporting neutral and acidic amino acids with moderate affinity except *AtAAP3* and *AtAAP5* which also transport basic amino acids (Fischer et al. 1995; Rentsch et al. 2007; Forsum et al. 2008). *AtAAP1* was found to be expressing in epidermis and was essential for amino acid import into embryo, *AtAAP8* also was found to be essential for uptake of amino acids into the endosperm during early seed development (Fischer et al. 1995; Hirner et al. 1998; Schmidt et al. 2007; Forsum et al. 2008; Sanders et al. 2009). *AtAAP2* and *AtAAP6* have been suggested to function in xylem-phloem transfer (Okumoto et al. 2002; Zhang et al. 2010). *AtAAP3* and *AtAAP5* expressing in the roots and root vasculature could be involved in amino acid uptake from the rhizosphere (Birnbaum et al. 2003; Okumoto et al. 2004). Furthermore, noteworthy members of the AAP subfamily from different species have been documented, including *S. tuberosum AAP1* (Koch et al. 2003), *PvAAP1* (Tan et al. 2008), *OsAAP8*, and *OsAAP15* (Zhao et al. 2012). The potential significance of *P. trichocarpa AAP11* in xylogenesis, possibly involving proline provision in *Populus*, has been suggested (Couturier et al. 2010).

Lysine-histidine transporters (LHTs) facilitate the transport of lysine, histidine, as well as neutral and acidic amino acids (Perchlik et al. 2014). AUX subfamilies are major auxin influx carriers, *AUX1* is found to be involved in regulating root gravitropism and root hair development. *AtANT1* is an aromatic and neutral amino acid transporter, ProT are Proline specific transporters were found be expressing in flowers and under salt-stress respectively in *Arabidopsis* (Chen et al. 2001; Birnbaum et al. 2003; Lee and Tegeder 2004; Swarup et al. 2004; Hirner et al. 2006; Foster et al. 2008; Zhang et al. 2010; Péret et al. 2012). Among APC family, various CAT subfamily members have been functionally characterized in *Arabidopsis*. *AtCAT5*, *AtCAT8*, *AtCAT1* and *AtCAT2* have been implicated in uptake of amino acids at the leaf margin, root, shoot meristems and tonoplasts to regulate soluble leaf amino acid concentration (Frommer et al. 1995; Su et al. 2004; Yang et al. 2014).

Though, functional characterization of *AAT* genes is limited to few plant species, genome wide identification studies have been carried out extensively and a varied number of genes were identified in *Arabidopsis*, rice, *Populus*, wheat, potato, rapeseed, soybean, strawberry, cotton, tomato and poplar (Rentsch et al. 2007; Zhao et al. 2012; Wu et al. 2015; Ma et al. 2016; Cheng et al. 2016; Wan et al. 2017; Tian et al. 2020; Liang et al. 2020; Yang et al. 2021; Omari 2021; Kong et al. 2022; Du et al. 2022). Genome wide identification of the gene families and annotation plays a pioneering step towards functional characterization of these genes in a plant species. When we investigate the list of species where AATs have been identified show a glaring lack of such studies in legumes. Until now *AAT* gene family was identified in *G. max* (Liang et al. 2020) and the current study adds *Phaseolus vulgaris* as the second to the list. Research on crucial gene families like AATs holds significance because, as transporters, they may play a vital role in transporting nutrients associated with Biological Nitrogen Fixation (BNF) and mycorrhization.. The biological nitrogen fixation initiates by the infection of legume host by bacteria rhizobia, resulting formation of root nodules. Bacteroids, the differentiated form of rhizobia reduces N_2_ to ammonia in the symbiosome which is later assimilated to glutamine, asparagine, and other amino acids (Atkins et al. 1982; White et al. 2007). While temperate legumes have aspargine as the major N transport form, tropical legumes produce ureides. Thus, produced amino acids are exported from nodules via xylem to the shoot or through phloem to the root for metabolism and growth (Pate et al. 1969, 1979; Atkins et al. 1982; Tegeder 2014). To transport these amino acids the role of AATs is indispensable. Moreover, arbuscular mycorrhizal (AM) fungi, besides transporting inorganic phosphate (Pi), also contribute to soil nutrient cycling by acquiring both organic and inorganic nitrogen (N) from the soil (Lanfranco et al. 2011). They absorb inorganic N compounds like ammonium, facilitated by various identified ammonium transporters (Gomez et al. 2009; Guether et al. 2009b; Kobae et al. 2010). However, besides *LHT1.2* in *Lotus japonicus* (Guether et al. 2011), no other AAT has been recognized for amino acid transport during AM symbiosis. Hence, in our present investigation we carried out an extensive analysis to identify AATs in *P. vulgaris* and classified them into 12 subfamilies based on their sequence and structural conservation as compared to *Arabidopsis* AATs. Further, we have researched into the differential expression patterns of *P. vulgaris* AATs using the data from the public data base and in house transcriptomic analysis.

## Materials and methods

### Identification of the AAT family members in *P. vulgaris*

*Phaseolus vulgaris* AAT sequences were identified based on the *Arabidopsis*, *Oryza sativa* and *Glycine max* AAT sequences. BLASTN and BLASTP search in Phytozome 13/*Phaseolus vulgaris* v2.1 genome database (https://phytozome-next.jgi.doe.gov/). In addition to Phytozome, Legume Information System (https://legumeinfo.org) genome databases was used to verify the AAT homologs. The conserved domains of the *P. vulgaris* AAT genes identified above were analyzed, and the genes that did not contain the PF01490 and PF00324 domains were removed. The nucleic acid and peptide sequences were retrieved from the online tool PhytoMine, accessible through the plant comparative genomics portal Phytozome 13, in preparation for subsequent analysis and annotation. Basic information on physico-chemical properties of AAT genes, including chromosome number, length of gene, CDs, peptide; isoelectric point (pI) and molecular weight (MW) was predicted through the Phytozome and ExPASy (https://web.expasy.org/protparam/). The subcellular localization of the complete protein sequences of AATs was predicted using the *in silico* tool Plant-mPLoc (version 2.0), available at http://www.csbio.sjtu.edu.cn/bioinf/plant-multi/.

### Chromosomal location of *Phaseolus vulgaris AAT* genes

The chromosomal localization of AAT gene family members was confirmed using the Legume Information System database (accessible at https://legumeinfo.org). Gene nomenclature followed the guidelines set forth by Quezada et al. (2019). Centromere locations and scales were determined based on the methods outlined by Fonsêca et al. (2010) and Wang et al. (2016), respectively.

### Phylogenetic analysis of *P. vulgaris* AAT superfamily genes

We utilized the complete amino acid sequences of AAT protein genes for conducting phylogenetic analysis. Initially, all obtained sequences (*A. thaliana*, *O. sativa*, *G. max* and *P. vulgaris*) were aligned using the Clustal Omega tool (https://www.ebi.ac.uk/Tools/msa/clustalo/) with default parameters. Using Molecular Evolutionary Genetics Analysis (MEGA 11), we performed phylogenetic analysis on the aligned sequences employing the neighbor-joining (NJ) method. The JTT + I + G substitution model was applied, along with 1000 bootstrap replicates and default parameters. The *P. vulgaris* AAT genes were classified into different groups according to the topology of the phylogenetic tree.

### Gene structure, conserved motif and promoter analysis of AATs

To visualize gene features, we utilized DNA sequences of AATs obtained from Phytozome 13. This included the structural information and positioning of exons, introns, and untranslated regions, which were reconstructed using the Gene Structure Display Server2.0 (GSDS2.0; http://gsds.gao-lab.org/Gsds_about.php) as described by Hu et al. (2015).

We utilized the Multiple Expectation Maximization for Motif Elicitation (MEME Suite 5.5.4) online program (https://meme-suite.org/meme/tools/meme) with specified parameters: number of repetitions = any, maximum number of motifs = 20, and optimal motif length range = 6 to 100 residues, to identify conserved motifs within the amino acid transporter (AAT) protein family in *P. vulgaris*. Following this, the more pertinent motifs underwent further scrutiny using the MOTIF Search interface (https://www.genome.jp/tools/motif/). Additionally, the respective identification numbers were acquired from the Pfam database (http://pfam.xfam.org/), streamlining the process of pinpointing conserved domains across various subfamilies.

The 2 kb DNA sequences preceding the start codon of amino acid transporter genes were retrieved from the Phytozome 13 database. Plant transcriptional regulatory elements (*cis*-elements) within the promoter sequences were analyzed using the PlantPAN 3.0 database (Chow et al. 2019) (http://plantpan.itps.ncku.edu.tw/plantpan4/index.html).

### Gene Ontology and TMH analysis

To gain functional insights into the identified AAT genes in *P. vulgaris*, Gene Ontology (GO) analysis was performed. The sequences of the identified AAT genes were submitted to the Blast2GO tool (https://www.blast2go.com/) for GO term annotation. The annotated GO terms provide information on the biological processes, molecular functions, and cellular components associated with each gene, and the results are represented graphically.

We employed the DeepTMHMM server (https://dtu.biolib.com/DeepTMHMM) to predict the topology of transmembrane domains, encompassing both alpha-helical and beta-barrel structures, within the amino acid transporter peptide sequences.

### Transcriptomic profiling and quantitative reverse transcription PCR analysis

Data on the differential expression of AAT genes in 24 tissues of *P. vulgaris* were obtained from both the PvGEA website (https://www.zhaolab.org/PvGEA/ )and Phytozome 13 (*P. vulgaris* v2.1). Additionally, data on AAT gene expression in arbuscular mycorrhiza (*Rhizophagus irregularis*) colonized roots was retrieved from our prior publication (Nanjareddy et al. 2017b). The construction of the heat map was performed using Fragments per Kilobase of Exon Model per Million Reads Mapped (FPKM) values for each AAT gene, with the analysis conducted in the *R* programming language.

To validate the RNA-seq data, we surface-sterilized *P. vulgaris* L. cv. Negro Jamapa seeds and germinated them as outlined by Nanjareddy et al. (2017a). In the experimental setup, two-day-old germinated seedlings were transferred into sterile vermiculite. Subsequently, inoculation with either *R. irregularis* or *R. tropici* was performed to assess *AAT* gene expression (Table S4) under mycorrhizal or root nodule symbiotic conditions following the procedure described by Nanjareddy et al. (2017b). Total RNA extraction and subsequent RT–qPCR analysis was performed in accordance with the protocol established by Arthikala et al. (2021). Relative gene expression levels were calculated using the 2^−ΔCT^ method, with ΔCT = CT_gene_ – CT_reference gene_. *P. vulgaris EF1*α and *IDE* were used as internal controls (Islas-Flores et al. 2011; Borges et al. 2012).

## Results

### Identification of *Pv*AAT protein orthologues in *P. vulgaris* genome

A total of 84 AATs were identified in *P. vulgaris* genome database using HMM model. We used *Arabidopsis*, *O. sativa* and *G. max* AAT sequences to conduct a BLASTN and BLASTP search in Phytozome 13 database. A total of 84 AAT gene family members were identified based on the presence of PF01490 and PF00324 domains and care was taken to made sure that the sequence was complete when compared to *Arabidopsis* AATs. In *Arabidopsis*, *O. sativa* and *G. max*, 63, 85 and 189 AATs have been reported respectively (Rentsch et al. 2007; Zhao et al. 2012; Kong et al. 2022). The AATs were classified into subfamilies depending on the sequence homology to other species and naming of the members of each subgroup was based on their appearance in the genome from the chromosomal short arm towards the long arm, starting from proximal to distal ends of the respective arms. We analyzed the physico chemical properties of *AAT* genes in *P. vulgaris* using online database. According to the assessment (Table 1), the lengths of *AAT* genes varied considerably with the largest protein CAT1 (641 aa) with the molecular weight (68.13 kD) and smallest CAT3 (278 aa) and the molecular weight (30.86 kD). Isoelectric point was highest in ProT3 (9.67) and lowest in ATLb6 (4.88) and prediction of subcellular localization showed most of the proteins in the plasma membrane and some proteins in the Golgi apparatus or chloroplast (Table S1).

**Table 1:** Physico-chemical properties of Amino Acid Transporter genes in *Phaseolus vulgaris*

Gene name^$^	Gene ID*	Chr. No.	Gene length, bp	Transcript length, bp	CDs length, bp	Peptide length, aa	pI	MW, kDa
Cationic amino acid transporter (CAT)								
CAT1	Phvul.001G104600	1	16035	2544	1923	641	6.43	68126.74
CAT2	Phvul.001G104700	1	7475	2418	1902	634	6.46	67120.50
CAT3	Phvul.002G001801	2	1423	1083	834	278	8.75	30865.58
CAT4	Phvul.002G252100	2	3819	2070	1809	603	6.92	65988.96
CAT5	Phvul.003G023101	3	11974	2125	1794	598	8.65	65414.52
CAT6	Phvul.003G225700	3	4473	2182	1728	576	8.41	63197.03
CAT7	Phvul.004G073100	4	12869	2316	1902	634	6.01	66862.88
CAT8	Phvul.007G023700	7	4935	2098	1752	584	8.83	62984.35
CAT9	Phvul.008G045400	8	5634	2176	1743	581	8.19	62169.85
CAT10	Phvul.009G217500	9	3488	2025	1767	589	8.58	64617.86
CAT11	Phvul.009G243100	9	1776	1776	1776	592	8.73	65545.49
CAT12	Phvul.009G243600	9	1773	1773	1773	591	8.73	65293.22
CAT13	Phvul.011G174000	11	2426	2426	1797	599	8.43	65187.90
Polyamine H+-symporter (PHS)								
PHS1	Phvul.002G052400	2	1863	1708	1410	470	5.45	52090.45
PHS2	Phvul.002G128000	2	2623	2294	1461	487	5.82	54265.11
PHS3	Phvul.002G235600	2	2486	2194	1431	477	5.82	52780.04
PHS4	Phvul.003G276300	3	1760	1760	1431	477	5.61	52938.39
PHS5	Phvul.008G177000	8	1515	1515	1092	364	9.05	40705.44
Amino acid/choline transporter (ACT)								
ACT1	Phvul.007G032900	7	5396	1760	1572	524	6.50	56707.05
ACT2	Phvul.007G032800	7	4826	1699	1563	521	8.64	56312.69
ACT3	Phvul.009G154100	9	3466	1687	1578	526	8.77	57803.86
Tyrosine-specific transporter (TTP)								
TTP1	Phvul.004G082300	4	4043	1972	1518	506	8.73	55156.33
Amino acid transporter-like protein (ATL)								
ATLa1	Phvul.001G248000	1	2976	2068	1311	437	8.22	47365.12
ATLa2	Phvul.004G045200	4	4753	2096	1404	468	8.45	50472.51
ATLa3	Phvul.004G045100	4	4424	2344	1392	464	6.71	49824.41
ATLa4	Phvul.005G179900	5	1443	1443	1443	481	5.44	52539.53
ATLa5	Phvul.008G024100	8	3594	2204	1401	467	7.00	50364.02
ATLa6	Phvul.008G219100	8	2360	1543	1302	434	8.31	46887.51
ATLa7	Phvul.008G219200	8	6435	1850	1338	446	8.20	48419.23
ATLa8	Phvul.009G198000	9	3621	1881	1386	462	6.16	49847.32
ATLb1	Phvul.001G123600	1	2669	1281	1281	427	5.02	47299.01
ATLb2	Phvul.001G263500	1	3138	1463	1425	475	8.88	51686.24
ATLb3	Phvul.002G156500	2	4997	1290	1290	430	5.14	47376.45
ATLb4	Phvul.006G071900	6	4997	1723	1320	440	7.59	47423.68
ATLb5	Phvul.007G097200	7	10785	2311	1632	544	5.59	58718.89
ATLb6	Phvul.007G256500	7	6425	1905	1563	521	4.88	56957.11
ATLb7	Phvul.008G040900	8	10133	1579	1389	463	8.88	50049.49
ATLb8	Phvul.008G040700	8	1473	1224	1224	408	8.89	44402.24
Aromatic and neutral amino acid transporter (ANT)								
ANT1	Phvul.001G199300	1	2882	1644	1221	407	6.34	43839.63
ANT2	Phvul.003G270400	3	1578	1578	1242	414	5.71	45183.65
Auxin transporter (AUX)								
AUX1	Phvul.001G241500	1	4043	2236	1431	477	8.79	53877.85
AUX2	Phvul.008G106300	8	6879	2538	1476	492	8.23	55333.68
AUX3	Phvul.008G225300	8	4904	2552	1440	480	8.59	54283.35
AUX4	Phvul.009G120700	9	6649	2473	2241	747	8.64	83631.55
AUX5	Phvul.009G122200	9	2856	1984	1398	466	8.76	52530.53
AUX6	Phvul.010G003600	10	6606	2208	1458	486	8.70	54792.03
AUX7	Phvul.011G034000	11	4379	1922	1458	486	9.02	54605.99
Amino acid permease (AAP)								
AAP1	Phvul.001G077000	1	5385	1900	1425	475	8.91	52652.43
AAP2	Phvul.001G076600	1	19554	1374	729	243	9.34	25865.28
AAP3	Phvul.001G071500	1	8779	1992	1452	484	8.64	53029.84
AAP4	Phvul.006G061100	6	4442	1933	1398	466	9.07	51363.26
AAP5	Phvul.006G061300	6	2853	1383	1383	461	8.87	51020.82
AAP6	Phvul.006G061500	6	4094	1956	1395	465	5.78	51045.85
AAP7	Phvul.006G061400	6	2700	1068	1068	356	9.46	39605.71
AAP8	Phvul.007G047400	7	4391	1536	1401	467	8.79	51104.87
AAP9	Phvul.008G282800	8	9224	2249	1386	462	8.43	51225.07
AAP10	Phvul.008G283000	8	3892	2233	1443	481	6.17	52228.60
AAP11	Phvul.009G112000	9	3546	2422	1410	470	9.50	51915.35
AAP12	Phvul.009G112100	9	5232	1617	1419	473	9.40	52229.48
AAP13	Phvul.009G113800	9	3886	1750	1449	483	8.88	53436.12
AAP14	Phvul.009G128500	9	5266	1754	1485	495	8.96	54469.45
AAP15	Phvul.009G153600	9	7436	2028	1461	487	8.64	53509.64
AAP16	Phvul.009G153700	9	6358	1784	1530	510	8.92	56025.20
C-aminobutyric acid transporter (GAT)								
GAT1	Phvul.002G296800	2	4976	1610	1359	453	8.93	49567.63
GAT2	Phvul.007G105500	7	2904	2017	1386	462	8.93	50459.37
GAT3	Phvul.007G177700	7	3835	2233	1308	436	8.76	48104.73
GAT4	Phvul.011G089600	11	3814	1592	1254	418	9.31	45936.18
GAT5	Phvul.011G089500	11	4917	1684	1371	457	8.83	50321.98
Proline transporter (ProT)								
ProT1	Phvul.001G239300	1	5290	1943	1329	443	9.14	48671.02
ProT2	Phvul.003G206600	3	3045	1609	1332	444	8.87	50008.44
ProT3	Phvul.008G227600	8	4171	1940	1152	384	9.67	42152.01
lysine and histidine transporter (LHT)								
LHT1	Phvul.001G259200	1	4611	1587	1587	529	9.19	59873.05
LHT2	Phvul.002G113000	2	4043	1946	1608	536	8.94	58510.62
LHT3	Phvul.002G176400	2	2042	1338	1338	446	8.85	49730.03
LHT4	Phvul.003G028400	3	3757	1766	1320	440	9.11	49116.74
LHT5	Phvul.003G208900	3	1868	1299	1299	433	8.91	48793.50
LHT6	Phvul.004G056700	4	6555	1663	1356	452	8.55	50725.82
LHT7	Phvul.005G001700	5	2488	1545	1329	443	9.11	49137.89
LHT8	Phvul.006G190600	6	4566	2113	1554	518	9.34	57111.86
LHT9	Phvul.007G103000	7	2783	1407	1287	429	9.00	47870.16
LHT10	Phvul.008G149700	8	3973	1347	1347	449	8.76	50295.27
LHT11	Phvul.008G149800	8	7809	1817	1392	464	8.10	52133.13
LHT12	Phvul.009G021300	9	4690	1822	1524	508	9.18	56000.34
LHT13	Phvul.011G025100	11	3924	1886	1503	501	9.08	54659.34

$ - Nomenclature based on the chromosomal location of the subfamily genes, * - Gene identification in Phytozome (https://phytozome.jgi.doe.gov), *bp* Base pairs, *CDS* Amino acid coding sequence, *aa* Amino acids, *pI* Isoelectric point, *MW* Molecular weight, *kDa* Kilodaltons.

### *Phaseolus vulgaris* AAT classification and chromosomal localization

**Fig. 1:**
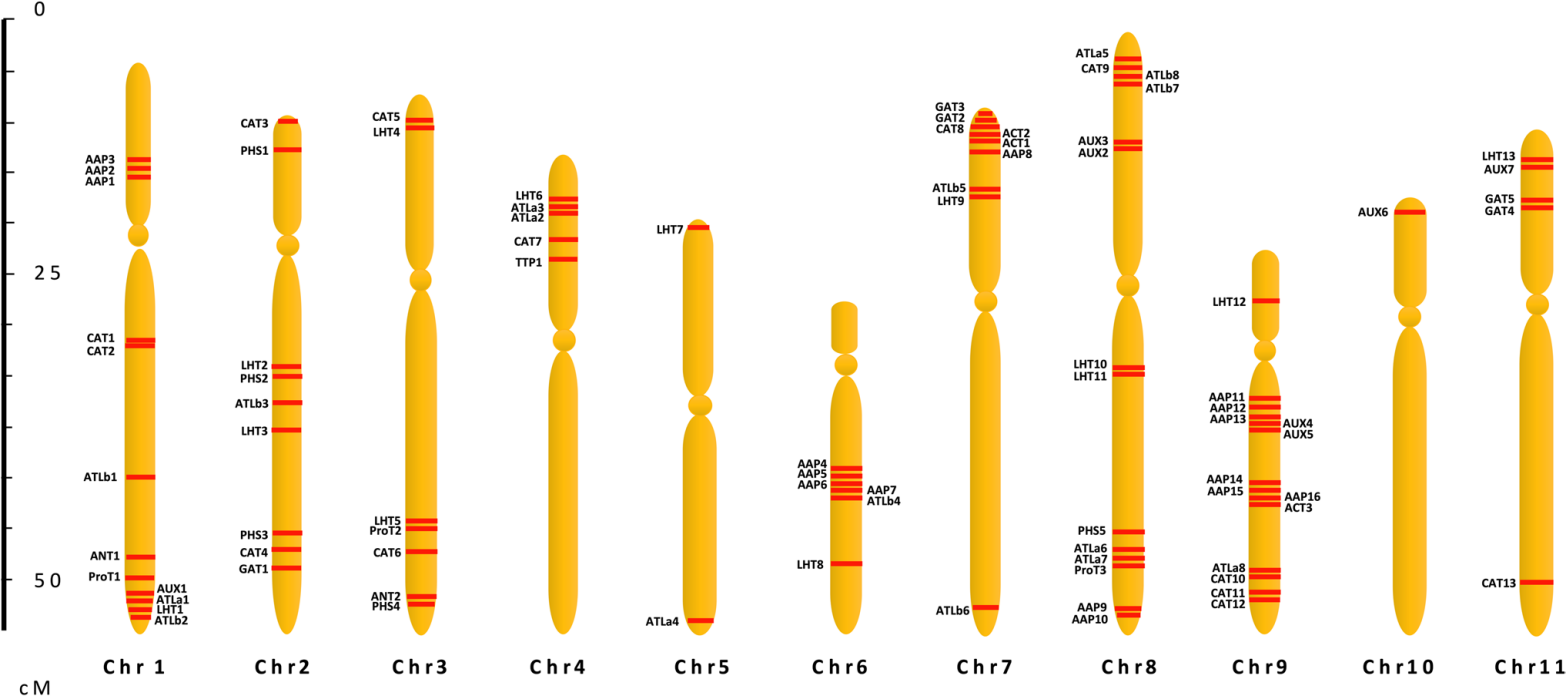
Chromosomal localization of Amino Acid Transporters (AAT) super family genes in *Phaseolus vulgaris*. The chromosomes are represented by the yellow colour and distributed numerically. The red bands highlight the putative *PvAAT* gene positions on the chromosomes. The scale is in megabases (Mb)

The superfamily of 84 AATs is classified into 12 subfamilies with an unequal distribution in each subfamily as follows, 13 genes in cationic amino acid transporters (CAT), 5 genes in the polyamineH+-symporters (PHS), 3 genes in amino acid/choline transporters (ACT), one gene of tyrosine specific transporter (TTP), 16 genes of amino acid transporter-like proteins (ATL) in turn classified into 2 subfamilies: 8 ATLa y 8 ATLb, 2 genes of aromatic and neutral amino acid transporter (ANT), 7 genes Auxin transporters (AUX), 16 genes of amino acid permeases (AAP) (Tabla 8), 5 genes of γ-aminobutyric acid (GABA) transporter (GAT), 3 genes de proline transporters and finally 13 genes of lysine/histidine transporter (LHT). The AATs in *P. vulgaris* were unevenly distributed among all the 11 chromosomes (Fig. 1). Chromosome 8 had a maximum of 14 genes followed by Chr1 with 12, Chr9 with 11, Chr2 and 7 with 9 genes each, Chr3 with 7 and Chr10 had a minimum of 1 gene. Most of the chromosomes showed accumulation of the genes towards the outer edges of the chromosomal arms except for the submetacentric chromosome Chr9 where the long arm had 13 genes spread out (Fig. 1).

### Phylogenetic analysis of AATs

**Fig. 2:**
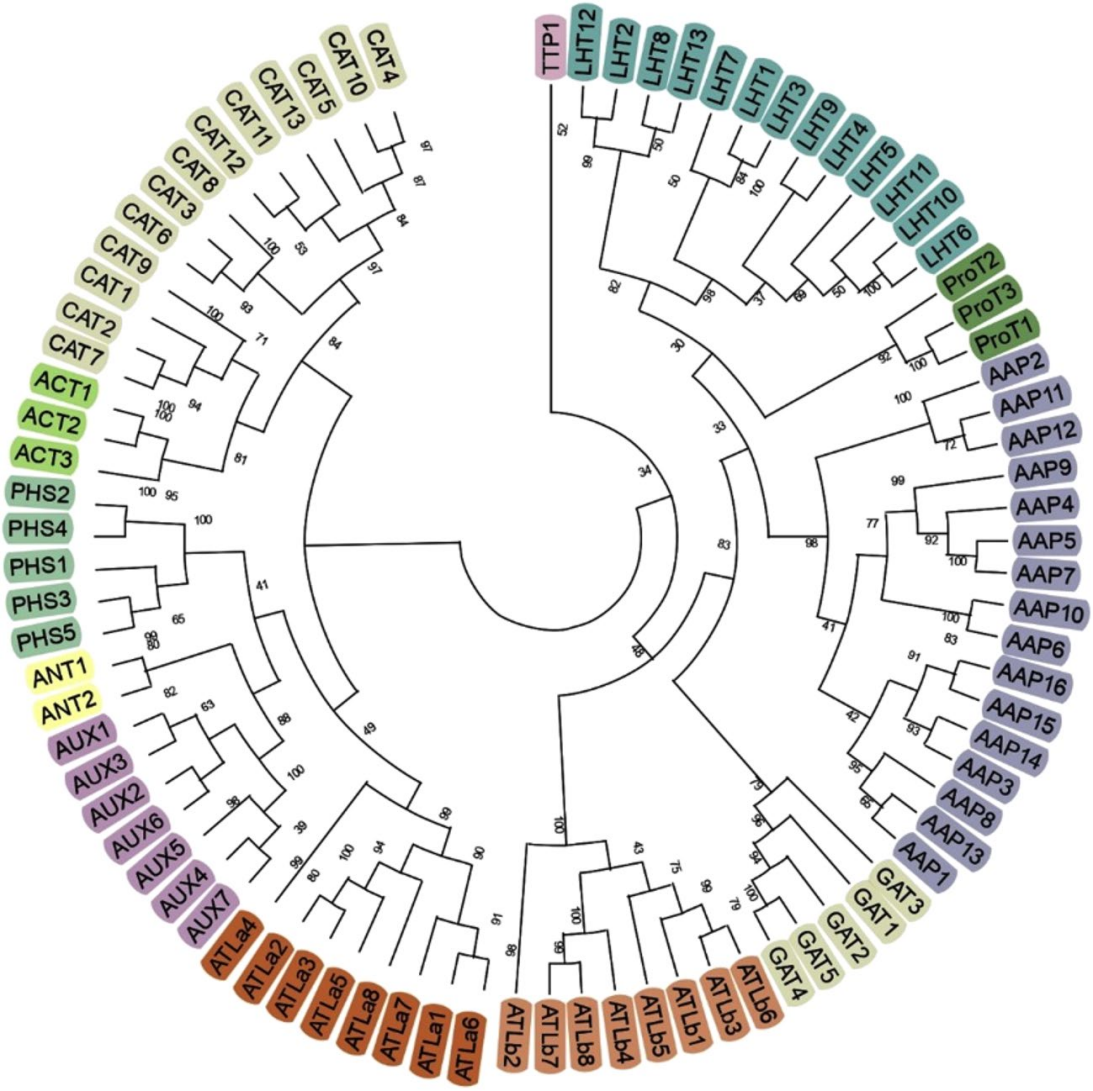
Phylogenetic analysis of the Amino Acid Transporters (AAT) superfamily in *Phaseolus vulgaris*. The amino acid sequences of 84 *AAT* genes identified in the Phytozome database. The phylogenetic tree was constructed using MEGA 11 software with the Neighbor-Joining tree method with 1000 bootstrap values. Subfamilies within the *AAT* superfamily are distinguished by different colors

To explore the evolutionary relationship of the AAT superfamily genes, a phylogenetic tree was constructed for 84 *P. vulgaris* AAT protein sequences. A phylogenetic tree developed using MEGA 11 with the neighbour-joining (NJ) method classified the AAT proteins into 12 clades (Fig. 2) which is like *Arabidopsis* and other plant species where the AAT superfamily has been identified and annotated. These 12 clades could be broadly classified into 2 families AAAP and APC family were, the AAAP family consists of 54 AATs, including seven distinct subfamilies: 8 genes in *ATLb*, 2 genes of aromatic and neutral amino acid transporter (*ANT*), 7 genes Auxin transporters (*AUX*), 16 genes of amino acid permeases (*AAP*), 5 genes of γ-aminobutyric acid (*GABA*) transporter (*GAT*), 3 genes de proline transporters (*ProT*) and finally 13 genes de lysine/histidine transporter (*LHT*). The *APC* family is comprised of 5 subfamilies including 30 AATs including 13 genes in cationic amino acid transporters (*CAT*), 5 genes in the polyamine H+-symporters (*PHS*), 3 genes in amino acid/choline transporters (*ACT*), one gene of tyrosine specific transporter (*TTP*), 8 genes of amino acid transporter-like proteins (*ATLa*) (Fig. 2).

Further, to establish evolutionary relationship and identify orthologous genes between monocots, dicots and legumes, non-legumes, a phylogenetic tree was developed using protein sequences from *A. thaliana*, *O. sativa*, *G. max* and *P. vulgaris* (Fig. S1). The combines phylogenetic tree developed the classification of the AAT proteins slightly different than what was seen in *P. vulgaris*. The classification of subfamilies was similar in all the species compared forming 12 clades indicating the formation of AATs before divergence of the monocots and dicots. There was a considerable variation in the total number of proteins in each of the subfamilies specifically, in AAPs where *P. vulgaris* has 16 genes, *Arabidopsis*, *O. sativa* and *G. max* genomes had 8, 19 and 34 genes each. Further total number of genes in APC subfamily are 25, 15, 35 and 30 genes in *O. sativa*, *Arabidopsis, G. max* and *P. vulgaris* respectively.

### Motif conservation and gene structure

**Fig. 3:**
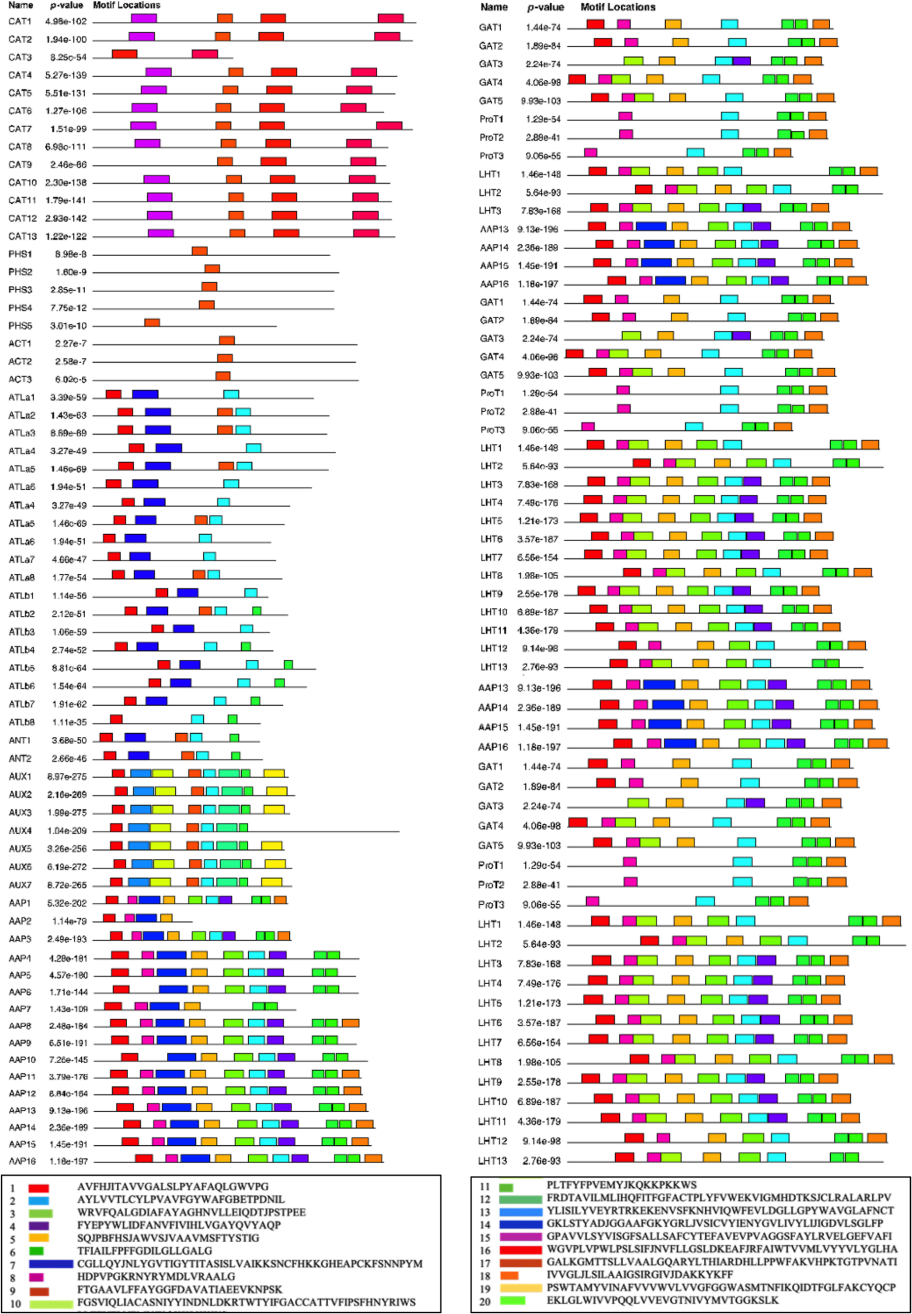
The conserved motifs identified through MEME-suite analysis of Amino Acid Transporter (AAT) proteins obtained from *Phaseolus vulgaris*. A total of 20 distinct motifs are identified across various AAT sub-families. Each color within the predicted protein domains represents a unique motif, providing insights into the structural and functional diversity of AAT proteins in *P. vulgaris*

MEME tools were used to identify motifs shared in AATs of *Phaseolus*. A total of 20 motifs were identified among both AAAP and APC families of AATs. The distribution of motifs in all *P. vulgaris* AATs are as shown in Fig. 3. Some of the motifs were well conserved and widely distributed among *P. vulgaris* AATs in AAAP family while some were specific to each subfamily. The frequent motif in most of the AATs was motif 2 followed by motif 1 and 6 which could be found in 7 subfamilies, all of which belonged to subfamilies ATL, ANT, AUX, AAP, GAT, LHT, ProT belonged to the family AAAP. Among AAAP subfamilies AAP had a characteristic motif 7, ATLa, ATLb had motif 14 and AUX had the motifs 10, 12, 13 and 19 restricted to the subfamily alone. Among APC subfamilies, CAT had characteristic motifs 15, 16 and 17, PHS and ACT had only motif 9. In general, the subfamilies in AAAP shared more motifs ranging from ATL with 4 motifs to LHT with 10 motifs. While APC family members had a maximum of 4 motifs in CAT and ATLa and ACT and PHS subfamilies had only one motif (Fig. 3).

**Fig. 4:**
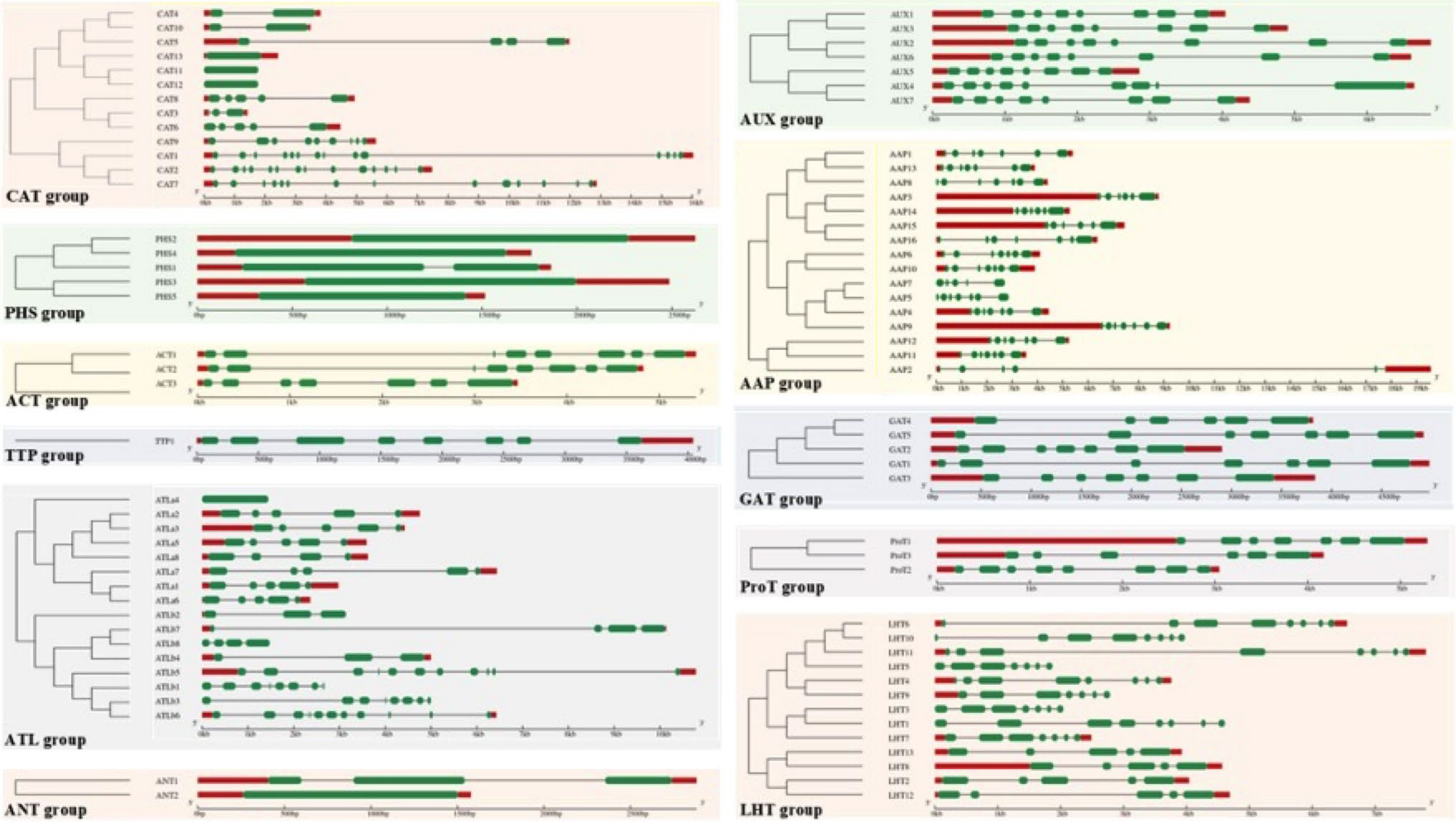
Gene structure analysis of the Amino Acid Transporters (AAT) superfamily genes in *Phaseolus vulgaris* organized by subfamilies. Coding sequences (CDs) and exons are represented by green bars, introns with black lines and untranslated regions (UTR's) in the ´upstream´ (5')/´downstream´ (3') direction with red boxes. The scale bar is shown at the bottom (Kb). Gene structure maps were drawn with the Gene Structure Display Server 2.0. The scale bar is shown at the bottom (Kb)

To understand the gene structure of AAT transporters in *P. vulgaris*, we compared the full-length cDNA sequences to the corresponding genomic DNA sequences using GSDS 2.0. Among all the *P. vulgaris* AATs, 9 genes did not have any introns, which included 4 genes of *PHS* subfamily, 3 genes from *CAT* subfamily and one gene each from *ATL* and *ANT* subfamilies. Unlike in *AAT* family genes in other species, the number of introns within the same subfamily varied greatly. Among *CAT* subfamily, the intron numbers varied from 1 to 13, *ATL* subfamily had 0-10 introns, *AAP* and *GAT* had 4-6, *LHT* from 4-7 introns (Fig. 4).

AATs being the membrane bound proteins, it was crucial to understand the distribution of transmembrane domains among the subfamilies. We developed the transmembrane domain structures using DeepTMHMM software. The results showed that CAT subfamily had lowest of 6 TMDs in CAT3 and a maximum of 15 TMDs were found in CAT7 and 8 with the remaining genes having 10-14 TMDs. PHS and ACT subfamily had 10-12 TMDs, TTP subfamily had 11, ATLa subfamily had 11-12, ATLb ranged between 6 to 11 and ANT, AUX had 10 TMDs each. The multiple sequence alignment of subfamilies showed highly conserved nature of TM domains in all the subfamilies (Fig. S2).

### *Cis –* acting elements identification and gene ontology

**Fig. 5:**
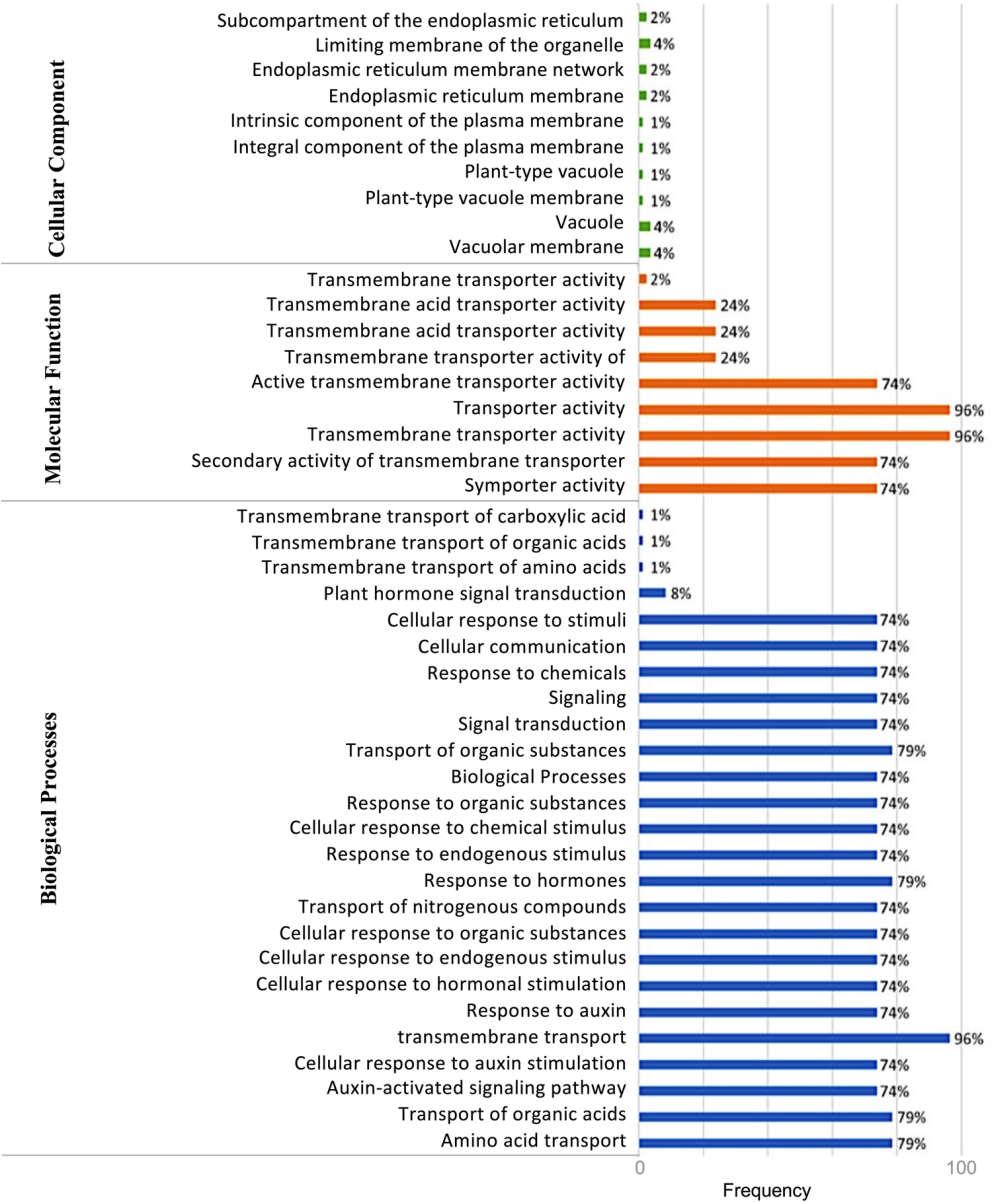
Gene Ontology enrichment analysis of cellular component, molecular function and biological processes for the members of *Phaseolus vulgaris AAT* genes. Bars indicates the frequency of genes with the same term

To identify the putative *cis*-element in the promoter region of the *AAT* genes, the first 2.0 kb of *AAT* genes of *cis*-elements was analyzed using the Plantpan database. The highest number of *cis*-elements were WRKY, AT-Hook, MYB and Homeodomain which are involved in regulation of gene expression, influence in biological processes such as growth, development, and response to abiotic and biotic stresses. Apart from these, several other *cis*-elements such as stress inducible AP2; ERF, lateral organ growth regulating B3, ABA-responsive element (ABRE) binding bZIP, plant growth and development and stress responsive C2H2, cell elongation regulating bHLH, calcium signal responsive CG-1 are found frequently. Further, ERFs involved in regulation of developmental processes in response to stimuli, and NAC, C2H2 and bHLH involved in the pathogen response, cell proliferation and development are some of the important cis - elements found on the promoter regions of AATs (Table S2). Eight of the identified *cis* elements, including AP2, ERF, bHLH, bZIP, C2H2, MYB/SANT, NAC, and WRKY, have previously been documented as specific to root nodule symbiosis (RNS). According to the GO molecular functional analysis and biological processes, 96% of them to be involved in activities related to transmembrane transporters involved in transport of amino acids, organic acids, nitrogenous compounds. Some are involved in response to various stimuli such as hormones, more specifically to auxins, organic compounds, chemicals etc. (Fig. 5).

### Expression analysis of *AAT* genes in different *Phaseolus vulgaris* tissues

**Fig. 6:**
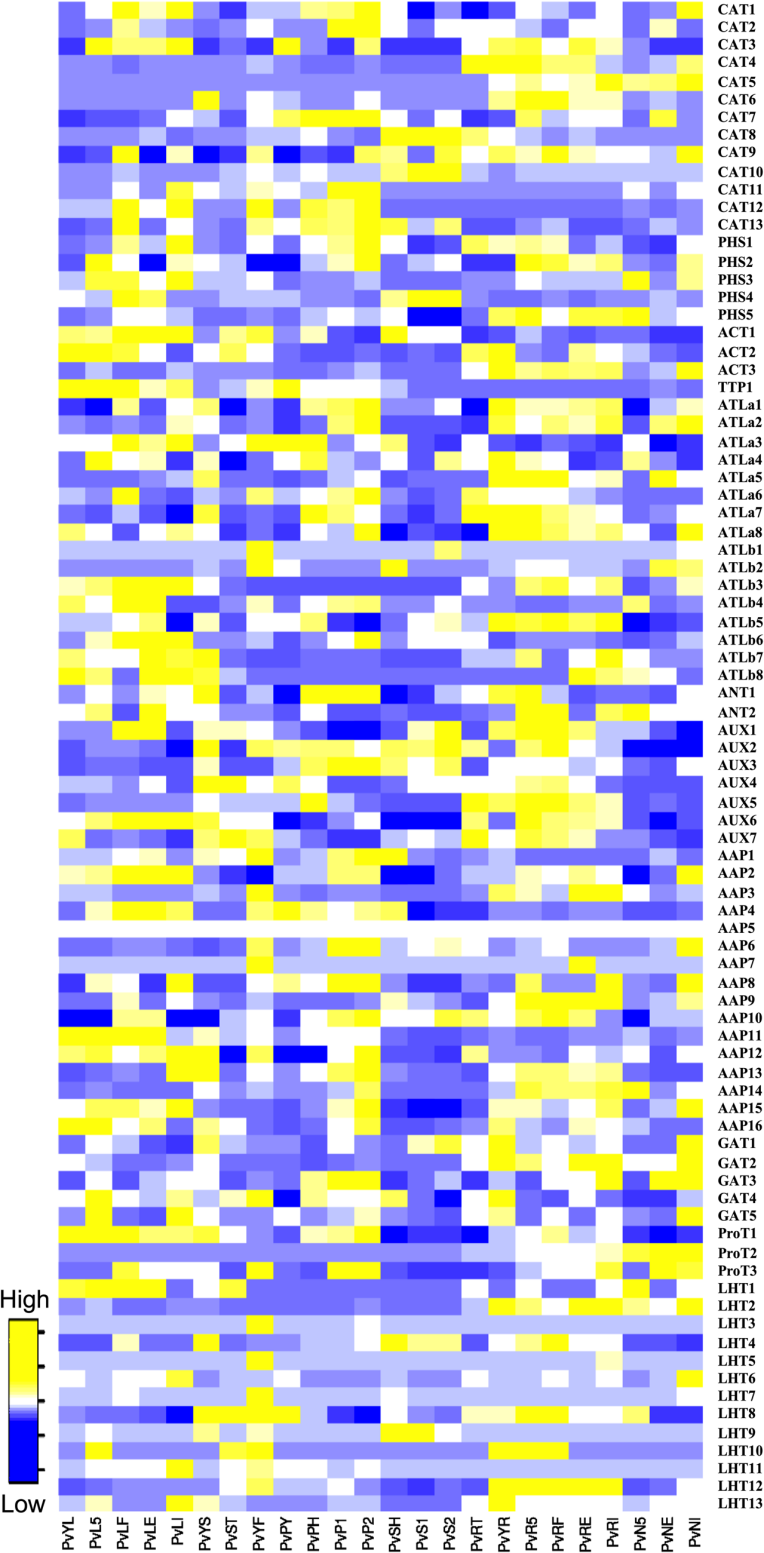
*In silico* expression profiles of *P. vulgaris AAT*s. Heat map expression profiles of *AAT* family genes in various tissues of *P. vulgaris*. Transcriptome data spanning diverse tissues (Table S3) were extracted from both Phytozome 13 (*P. vulgaris* v2.1) and the *P. vulgaris* Gene Expression Atlas (PvGEA). The generation of the heat map was facilitated by utilizing the Fragments per Kilobase of Exon Model per Million Reads Mapped (FPKM) values for each *AAT* gene, a process executed through the programming language *R*

To principally investigate the probable gene function of AATs in various *Phaseolus* tissues, the tissue−specific expression data of AATs were first downloaded from the PvGEA: Common bean Gene expression atlas and Network analysis, which includes the transcriptional profiles of different *P. vulgaris* tissues (Table S3) including young leaves, mature leaves, roots, flower, pods, seeds and nodules. As it is evident from the heat map (Fig. 6) most of the AATs had low to very low expression levels in all the tissues studied. Among APC family, CAT subfamily genes had very low expression except for *CAT8* and *CAT10* which showed high expression specifically in seeds, *CAT7* was found to be expressing in pods. Among PHS subfamily, *PHS3* had very high expression in leaves derived from *Rhizobium* inoculated plants. ACT subfamily showed low expression in leaf tissues, *ACT2* specifically showed very high expression in young leaf and *ACT3* in young roots. *ANT1* showed pleotropic expression in all the tissues listed. *ATLa5* gene showed elevated expression in leaves and young root, while other *ATLa* and *ATLb* genes didn’t show significant expression. *AAP1* and *AUX3* were the genes showing positive expression in most of the tissues studied. *AUX* genes were positively regulated in pods and ProT subfamily genes expressed mostly in young shoots, pods, and leaves. *LHT2* and *LHT13* showed very high expression in young root and leaves respectively. GAT subfamily had very high expression root tissues (Fig. 6).

### Expression analysis of *AAT* genes during rhizobial and mycorrhizal symbiosis

**Fig. 7:**
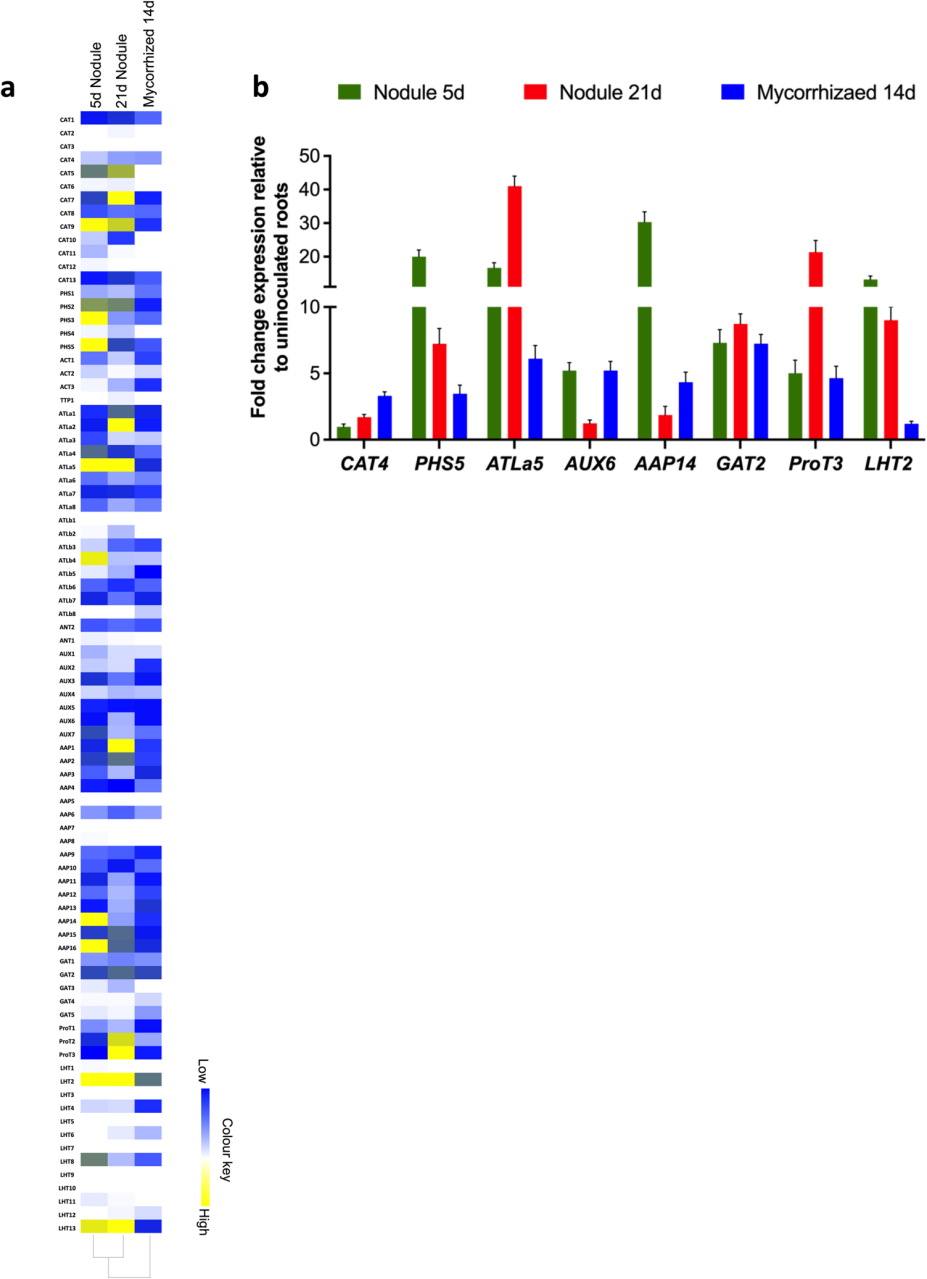
Analysis of *AAT* gene expression in response to root symbionts in *P. vulgaris*. **a)** Heat maps showing the differencial *AAT* gene expression patterns specific to rhizobial and mycorrhizal colonization nodules and roots, respectively. Colour bar shows the fold-change range, with yellow and blue representing upregulation and downregulation, respectively. **b)** Validation of the transcriptomic data by RT-qPCR analysis. The expression of 8 AAT genes in nodules, and mycorrhized roots was quantified by RT-qPCR. The data are the averages of three biological replicates (*n* > 9). Error bars represent means ± Standard error mean (SEM)

Further, analysis of AAT gene expression specifically in *Rhizobium* inoculated and mycorrhiza colonized *P. vulgaris* tissues was done using the PvGEA: Common bean Gene expression atlas and Network analysis database and the data from our previous transcriptomic analysis (Nanjareddy et al. 2017b). Gene expression data for root nodule symbiosis were downloaded from the PvGEA: Common bean Gene expression atlas and Network analysis for 5 days post inoculation (dpi) and 21 dpi. The data revealed very interesting findings as presented in shown in the Fig. 7a. At 5 dpi, among 84 AATs, 36 genes had no, or very low expression (< 2) and 10 genes were highly expressed. However, *AAP14* (60.9) and *LHT2* had the highest expression (136.3) when compared to any other gene. At 21 dpi, 49 genes had no, or very low expression (< 2) which amounts to more than half of *ATT* genes. The highest gene expression at 21 dpi was observed in *ATLa5* (108.6), followed by *LHT2* (80.3) among the top 9 expressed genes. To analyze the differential expression of AATs under mycorrhizal symbiotic condition we used the transcriptomic analysis previously published from our group (Fig. 7a). The data shows that *LHT2* as very highly expressed in comparison to all the other AATs followed by *ATLa5*, *AAP3*, *AAP13*, *AAP16* and *GAT2*. Among all the AATs, 33 genes did not or very low express (< 2) in mycorrhizal symbiotic condition.

Subsequently, RT−qPCR was performed to validate the expression data retrieved from PvGEA and RNA-seq in *P. vulgaris*. The transcript accumulation of random eight genes viz., *CAT4*, *PHS5*, *ATLa5*, *AUX6*, *AAP14*, *GAT2*, *ProT3* and *LHT2* were analyzed at 5 dpi and 21 dpi and 14day mycorrhizal colonized root tissues of *P. vulgaris* (Fig. 7b). The expression of the chosen genes was consistent with the RNA-seq data (Fig. 7a, b). Taken together, we propose that *LHT2* and *ATLa5* might be involved in root nodule symbiosis both at early and late stages and in mycorrhizal symbiosis. Further, AAP subfamily genes could be significant to carry out functional genomic studies during *P. vulgaris* symbiotic associations.

## Discussion

Amino acid transporters in plants are one of the largest gene families playing crucial roles during seed germination, growth and development of the plants, seed formation, biotic and abiotic stress through transport of amino acids (Yao et al. 2020). The inorganic salts of nitrogen acquired from the soil solution, these compounds are incorporated into amino acids in root and mature leaves in all the plant species. In legumes, through biological nitrogen fixation a variety of amino acids are synthesized and are transported to the sink organs through xylem and phloem. In any of these scenarios the role of amino acid transporters is indispensable. Though there has been extensive research on genome wide identification of AATs in plants, there is limited research in legumes. Herein, we identified and characterized AAT gene family members in *P. vulgaris* through genome wide analysis and differential gene expression in *P. vulgaris* tissues, finally AAT expression pattern in root nodule and mycorrhizal symbiotic condition.

AAT gene family in plants is classified as AAAP and APC families, with 54 and 30 genes under each family. AAAP family is further divided into 7 subfamilies, ATLb, ANT, AUX, AAP, GAT, ProT and LHT. APC family is divided into CAT, PHS, ACT, TTP and ATLa subfamilies. The characteristic motifs for identification of AAT gene family members were PF01490 (Aa_trans) and PF00324 (Aa_permease). Studies published so far have identified a varied number of AAT gene family members such as *Arabidopsis* (63), rice (85), wheat (296), potato (72), rapeseed (203), soybean (189), strawberry (45), cotton (*Gossypium hirsutum* - 190, *G. barbadense* - 190, *G. arboretum* - 101, and *G. raimondii* - 94), tomato (88) and poplar (83) (Rentsch et al. 2007; Zhao et al. 2012; Wu et al. 2015; Cheng et al. 2016; Ma et al. 2016; Wan et al. 2017; Liang et al. 2020; Tian et al. 2020; Yang et al. 2021; Omari 2021; Kong et al. 2022; Du et al. 2022). In the current study we have identified 84 AAT genes in *P. vulgaris* which is like rice, tomato and poplar. These genes were found to be distributed on all the 11 chromosomes of the *P. vulgaris* genome however, a characteristic clustering of genes towards the outer edges of most of the chromosomes was noticed. Phylogenetic relationships with orthologs in comparison to *Arabidopsis* and rice were useful to identify the genes in subfamilies. The major division of the AATs in *P. vulgaris* was into 2 families as AAAP and APC which were further classified into 12 clades conserving the major clade structure as in other species. The number of genes in each subfamily varied as compared to other.

A total of 20 conserved motifs were identified in *P. vulgaris* AAT family. Among these, motif 1, 2 and 6 were found in most of the subfamilies belonging to AAAP subfamily. Among APC subfamilies, the motif conservation was rather specific to subfamilies than to the group of subfamilies under APCs. Gene structure analysis plays a crucial role in understanding the functional role of the genes. Gene family evolution is well represented through variation in gene structure (Javelle et al. 2011; Du et al. 2012; Hudson and Hudson 2015). Our findings show that multiple AAT genes were devoid of introns. The feature is common to other AAT genes in *Arabidopsis*, rice and soyabean. In summary, most members of the subfamilies shared similar intron/exon structures and gene length. Such conservation of the gene structure in subfamilies imply evolutionary relationships among them. Reports suggest that either complete absence or presence of shorter and fewer introns could be an indication of genes that are effectively expressed under stressful conditions (Heyn et al. 2015). These findings imply a probable rapid induction of *ANT2*, *ATLa4*, CAT11-13 and PHS subfamily genes to stress conditions. Further, analysis of trans membrane domains revealed a highly conserved nature of TMDs in each subfamily and this was consistent with the other species compared.

An analysis of *cis*-elements in the promoter region of *Phaseolus* AAT transporters showed presence of many *cis*-elements such as WRKY, AT-HOOK and MYB involved in growth, development, and abiotic and biotic stress response. Other frequently occurring *cis*-elements were ABA responsive (ABRE), cell elongation and cell proliferation implying the important role played by the amino acid transporters majorly in growth and development related aspects of *P. vulgaris*. Gene ontology studies further confirmed the biological role of ATTs as transporters involved in transport of amino acids, nitrogenous compounds among others.

When we analyzed the expression pattern of AATs in various tissues of *P. vulgaris*, we found most of the CAT subfamily genes expressing in shoot tissues, PHS showed very low expression in most of the tissues and PHS genes expressed very high either in roots or leaf tissues. Among AAAP subfamilies, *ANT1* expressed in all the tissues, *AUX* genes had very low expression in most of the tissues, GATs mostly expressed in roots, *ProT* genes highly expressed in leaves and pods, *LHT*, *AAP* and *ATL* were the least expressed genes in *Phaseolus* tissues. In contrast, studies on rice revealed LHT1 expression in roots, leaves, and flowers (Wang et al. 2019). Expression analysis of *AAP1* in *Vicia faba*, *P. vulgaris*, and *Pisum sativum* indicated specificity to cotyledons, while *V. faba AAP3* was expressed in roots, shoots, and pods (Tegeder et al. 2000; Miranda et al. 2001; Tan et al. 2008).

The significance of *AAT* gene expression in root nodule symbiotic conditions is noteworthy, given the role in biological nitrogen fixation. Limited studies have explored the involvement of AATs in symbiotic processes. Our analysis focused on 5 dpi and 21 dpi, representing early and late stages of RNS. Herein, our analysis showed *LHT2* as a gene with high expression at 5 dpi and 21 dpi during rhizobial symbiosis and 3 weeks post inoculation mycorrhizal symbiosis, followed by *ATLa5* as a next highly expressed gene. Under rhizobial symbiotic conditions, a total of 37 genes and 48 genes did not show positive regulation at 5 dpi and 21 dpi respectively. Similarly, 20 *AAT* genes were unresponsive under mycorrhizal symbiosis. In *P. sativum* PsAAP6 is reported to be a key player in nitrogen retrieval from the apoplasm into inner cortex cells for nodule export (Garneau et al. 2018) Response of *AAT* gene expression to rhizobial symbiosis is crucial to understand as during biological nitrogen fixation bacteroids, the differentiated rhizobia reduce N_2_ to ammonia and is later assimilated to glutamine, asparagine, and other amino acids or as ureides (Atkins et al. 1982; White et al. 2007). On the other hand, arbuscular mycorrhizal (AM) fungi can acquire both inorganic and organic nitrogen (N), transporting them through arginine from the extra- to the intraradical mycelium where N is transferred to the plant as inorganic N compounds such as ammonium. Previous studies identified altered expression of various amino acid transporters (AATs) in *Lotus japonicus* mycorrhizal roots through microarray analysis (Guether et al. 2009a) and functional characterization of *LHT1.2* in *L. japonicus* had demonstrated its role in intricate mechanisms for amino acid reuptake and recycling, specifically in mycorrhizal roots (Guether et al. 2011). While the exploration of amino acid transporters (AATs) in understanding their role in symbiotic associations has been limited, genome-wide identification studies could establish a crucial foundation for selecting candidate genes for further functional characterization.

### Supplementary Information

Supplementary file 1 **Fig. S1** Phylogenetic Analysis of the Amino Acid Transporters (AAT) Superfamily in *Arabidopsis thaliana*, *Oryza sativa*, *Glycine max*, *Phaseolus vulgaris*. The amino acid sequences of 63, 85, 189 and 84 AAT genes identified in the Phytozome database respectively for *A. thaliana*, *O. sativa*, *G. max*, *P. vulgari*. The phylogenetic tree was constructed using MEGA 11 software with the Neighbor-Joining tree method with 1000 bootstrap values. (PDF 1408 kb)

Supplementary file 2 **Fig. S2** Transmembrane topology models applied to *Pv*AAT superfamily. DeepTMHMM analyses of the protein sequences. Pink regions indicate putative transmembrane domains with the relative probability of each indicated on the Y-axis. Yellow and blue regions correspond to predicted extracellular and intracellular segments respectively (PDF 2900 kb)

Supplementary file 3 **Table S1** The subcellular localization of *AAT* genes segregated according to the subfamilies using *in silico* tool Plant-mPLoc (version 2.0) (http://www.csbio.sjtu.edu.cn/bioinf/plant-multi/). **Table S2** *In silico* promoter analyses of *PvAAT* genes by PlantPAN3.0 (XLSX 30 kb)

Supplementary file 4 **Table S3** The *Phaseolus vulgaris* tissues selected for expression analysis (PDF 77 kb)

Supplementary file 5 **Table S4** Primer sequences of *Phaseolus vulgaris* genes used to perform quantitative RT-PCR (PDF 29 kb)

## Data Availability

Not applicable
