# Supplementary material for: Genome-wide identification and comparative analysis of the Amino Acid Transporter (AAT) gene family and their roles during Phaseolus vulgaris symbioses
Source: Funct Integr Genomics. 2024 Mar 2;24(2):47. doi: 10.1007/s10142-024-01331-0 (PMC10908646; doi:10.1007/s10142-024-01331-0)
Supplement: Supplementary file 1 — Fig. S1 Phylogenetic Analysis of the Amino Acid Transporters (AAT) Superfamily in Arabidopsis thaliana, Oryza sativa, Glycine max, Phaseolus vulgaris. The amino acid sequences of 63, 85, 189 and 84 AAT genes identified in the Phytozome database respectively for A. thaliana, O. sativa, G. max, P. vulgari. The phylogenetic tree was constructed using MEGA 11 software with the Neighbor-Joining tree method with 1000 bootstrap values. (PDF 1408 kb) [file 10142_2024_1331_MOESM1_ESM.pdf]

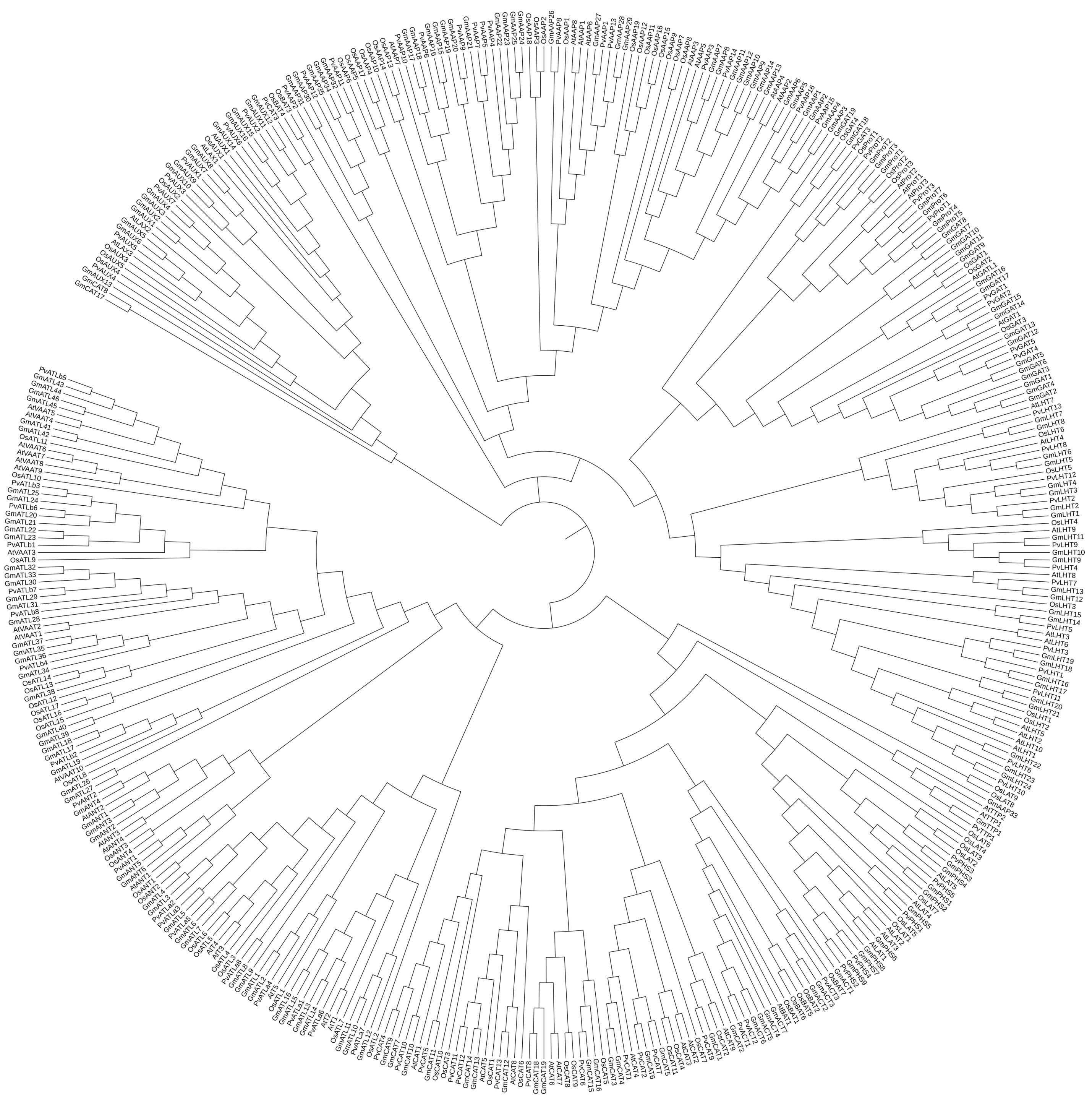

Figure S1 Phylogenetic Analysis of the Amino Acid Transporters (AAT) Superfamily in *Arabidopsis thaliana*, *Oryza sativa*, *Glycine max*, *Phaseolus vulgaris*. The amino acid sequences of 63, 85, 189 and 84 AAT genes identified in the Phytozome database respectively for *A. thaliana*, *O. sativa*, *G. max*, *P. vulgari*. The phylogenetic tree was constructed using MEGA 11 software with the Neighbor-Joining tree method with 1000 bootstrap values.
