# Supplementary material for: Genome-wide identification and comparative analysis of the Amino Acid Transporter (AAT) gene family and their roles during Phaseolus vulgaris symbioses
Source: Funct Integr Genomics. 2024 Mar 2;24(2):47. doi: 10.1007/s10142-024-01331-0 (PMC10908646; doi:10.1007/s10142-024-01331-0)
Supplement: Supplementary file 2 — Fig. S2 Transmembrane topology models applied to PvAAT superfamily. DeepTMHMM analyses of the protein sequences. Pink regions indicate putative transmembrane domains with the relative probability of each indicated on the Y-axis. Yellow and blue regions correspond to predicted extracellular and intracellular segments respectively (PDF 2900 kb) [file 10142_2024_1331_MOESM2_ESM.pdf]

CAT group

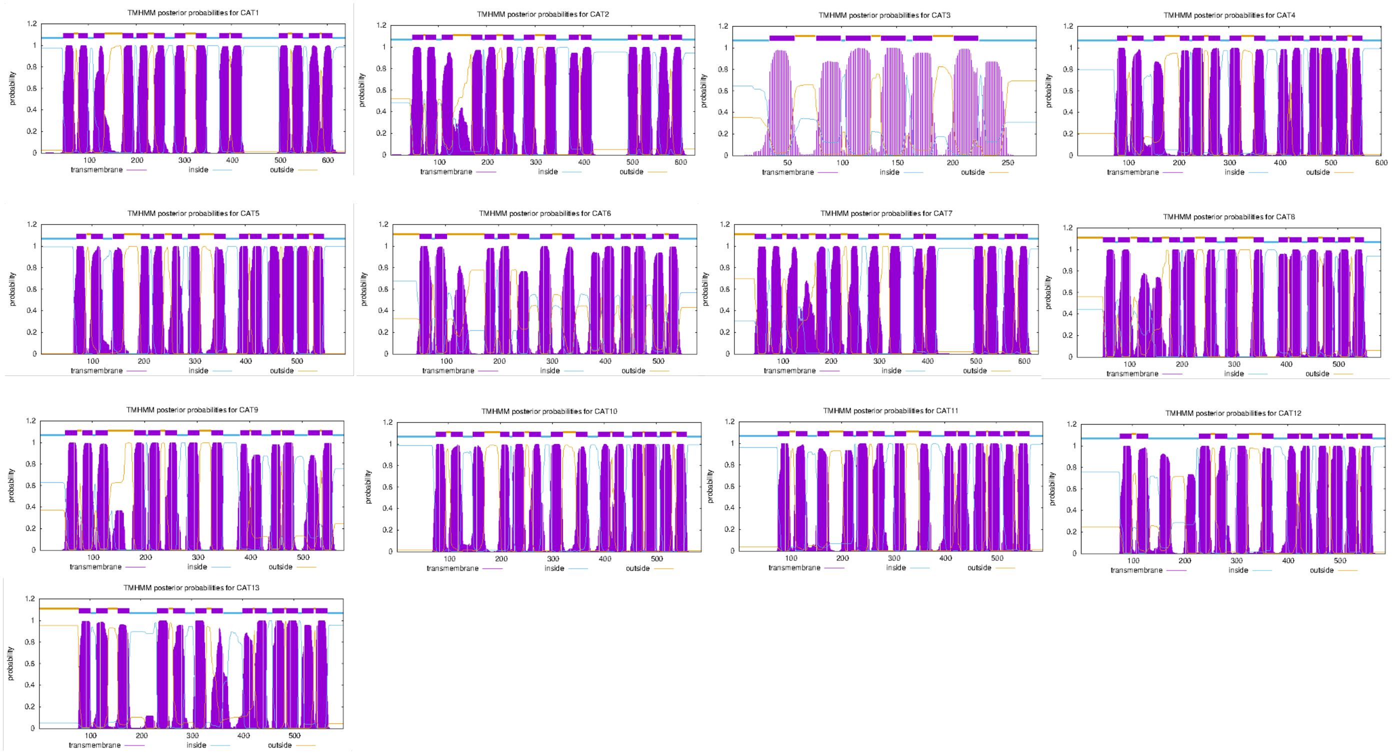

PHS group

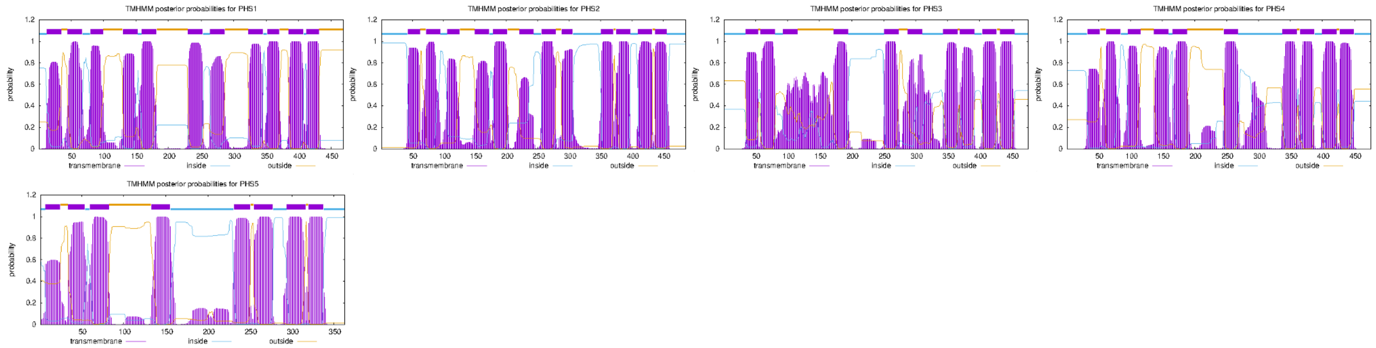

ACT group

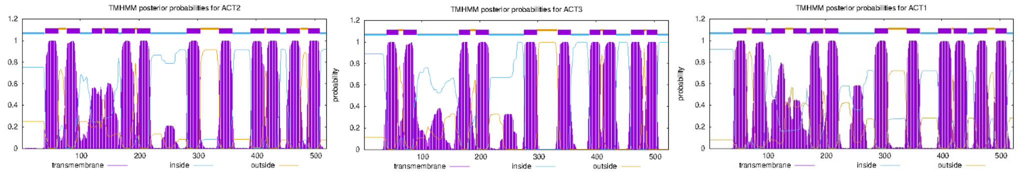

TTP group

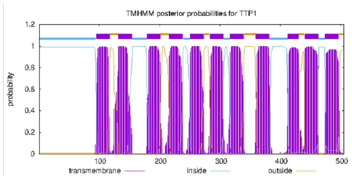

ATLa/ATLb group

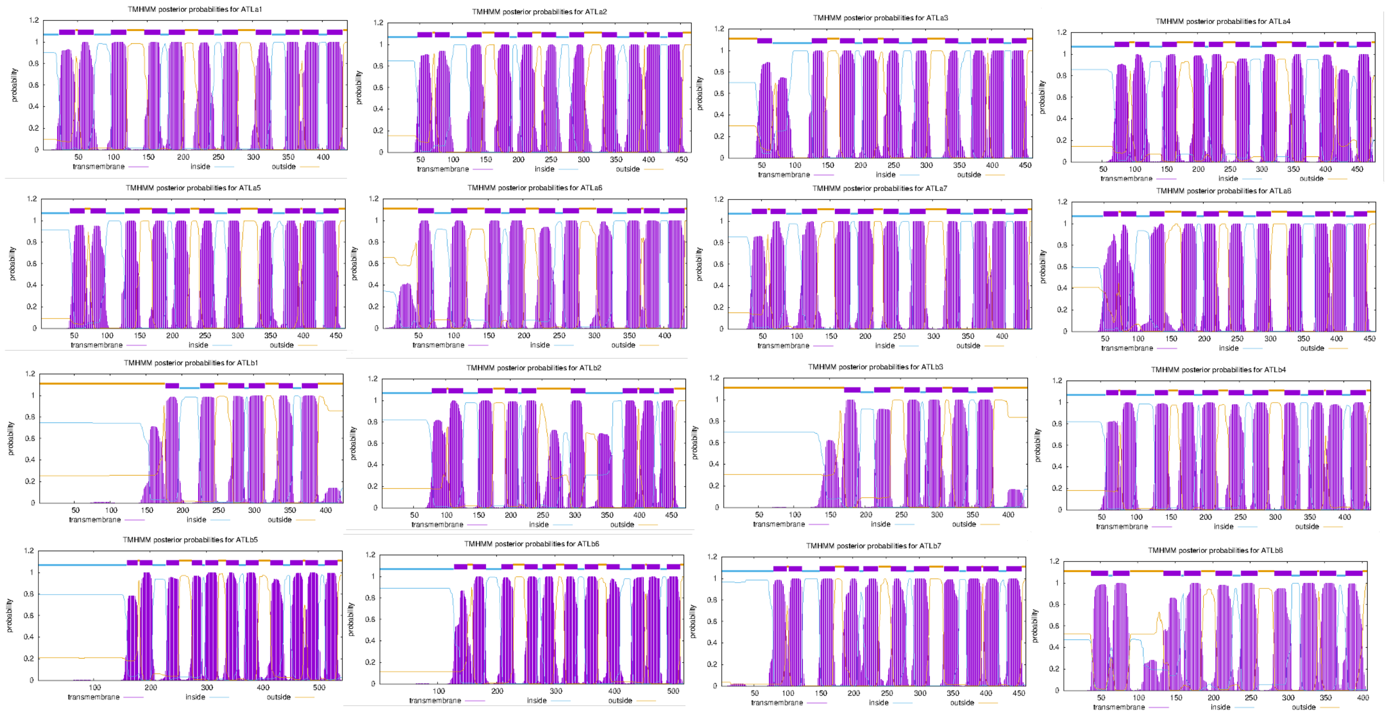

ANT group

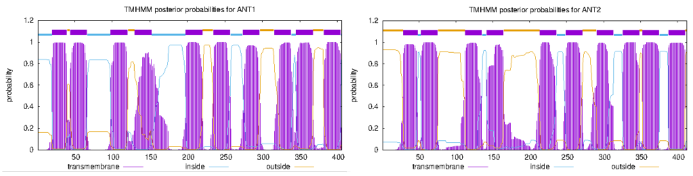

AUX group

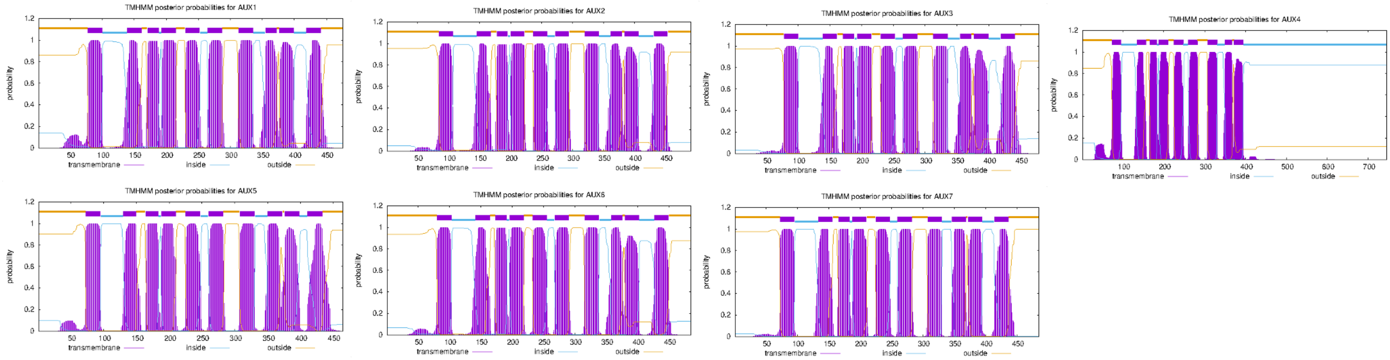

AAP group

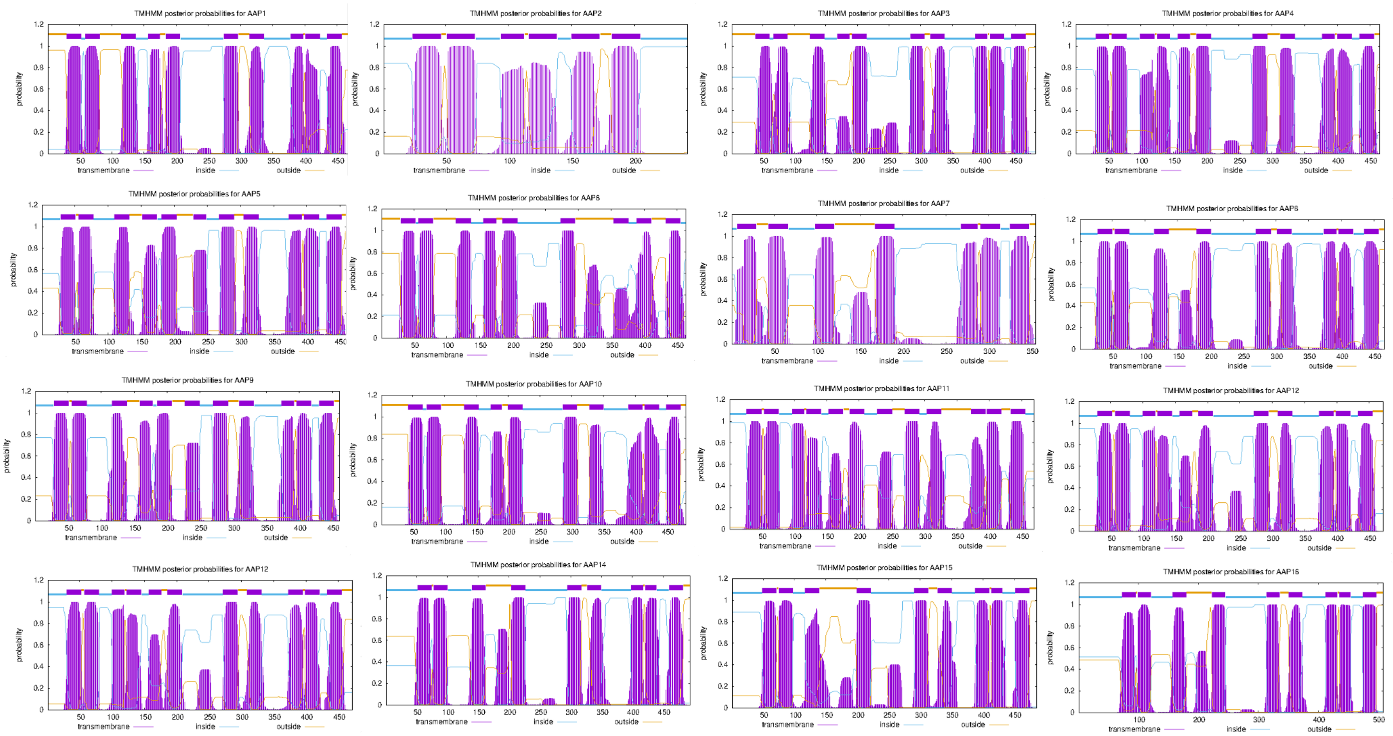

GAT group

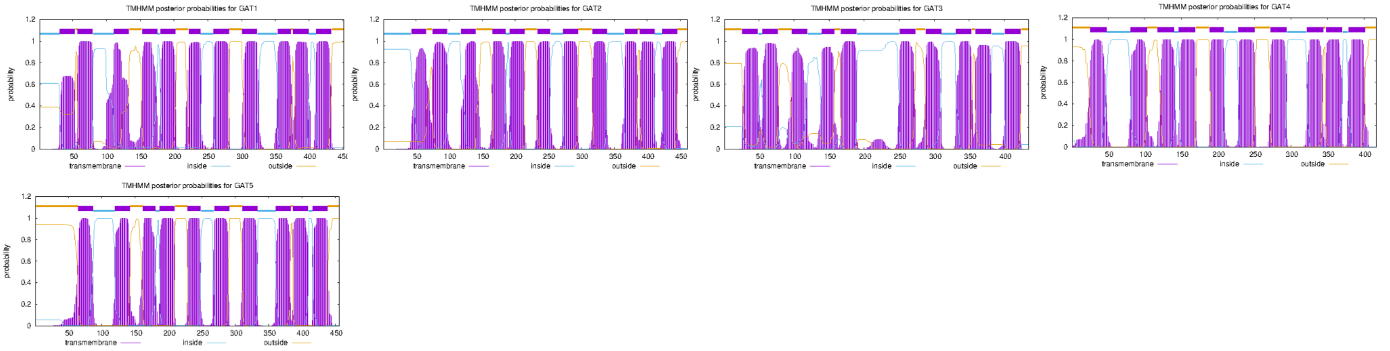

ProT group

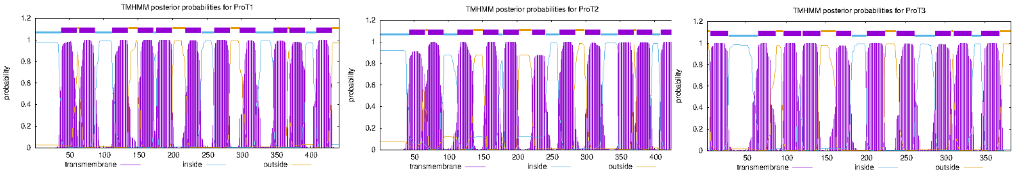

LHT group

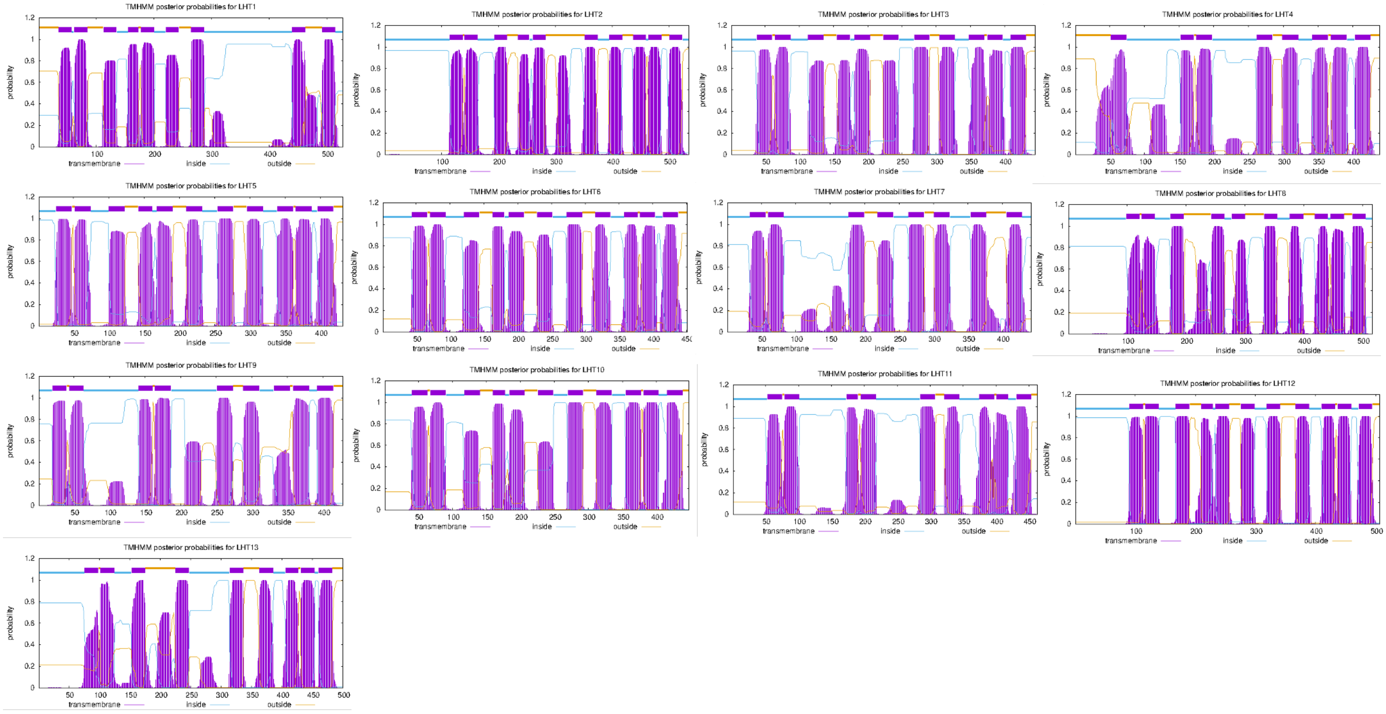

Figure S2. Transmembrane topology models applied to PvAAT superfamily. DeepTMHMM analyses of the protein sequences. Pink regions indicate putative transmembrane domains with the relative probability of each indicated on the Y-axis. Yellow and blue regions correspond to predicted extracellular and intracellular segments respectively.
