# Supplementary material for: Genome-wide identification and comparative analysis of the Amino Acid Transporter (AAT) gene family and their roles during Phaseolus vulgaris symbioses
Source: Funct Integr Genomics. 2024 Mar 2;24(2):47. doi: 10.1007/s10142-024-01331-0 (PMC10908646; doi:10.1007/s10142-024-01331-0)
Supplement: Supplementary file 4 — Table S3 The Phaseolus vulgaris tissues selected for expression analysis (PDF 77 kb) [file 10142_2024_1331_MOESM4_ESM.pdf]

Table S3: The *Phaseolus vulgaris* tissues selected for expression analysis.

Name	Tissue type[#]
**PvYL**	Tissue of the second trifoliate leaf completely expanded, in plants provided with fertilizer.
**PL5**	Leaf tissue, collected 5 days after plants were inoculated with effective Rhizobium.
**PvLF**	Leaf tissue from fertilized plants, collected 21 days after the plants were inoculated with Rhizobium.
**PvLE**	Leaf tissue, collected 21 days after plants were inoculated with effective Rhizobium.
**PvLI**	Leaf tissue, collected 21 days after plants were inoculated with ineffective Rhizobium.
**PvYS**	Stem internodes above cotyledon, collected in the second trifoliate stage.
**PvST:**	Shoot tip, including the apical meristem, collected in the second trifoliate stage.
**PvFY**	Young flowers, collected before flora emergence.
**PvPY:**	Young pods, harvested 1 to 4 days after floral senescence. The sample includes developing embryos in the globular stage.
**PvPH:**	Pods approximately 9 cm long, associated with seeds at the heart stage (pod only).
**PvP1**	Pods between 10 and 11 cm long, associated with stage 1 seeds (pod only).
**PvP2**	Pods between 12 and 13 cm long, associated with stage seeds (pod only).
**PvSH**	Heart stage seeds, between 3 and 4 mm wide and approximately 7 mg.
**PvS1**	Stage 1 seeds, between 6 and 7 mm wide and approximately 50 mg.
**PvS2**	Stage 2 seeds, between 8 and 10 mm wide and between 140 and 150 mg.
**PvRT**	Root tips, 0.5 cm of tissue, collected from fertilized plants in the second trifoliate stage of development.
**PvYR**	Whole roots, including root tips, collected at the second trifoliate stage of development.
**PvR5**	Whole roots separated from 5 day old pre-fixing nodules.
**PvRF**	Whole roots from fertilized plants collected at the same time as RE and RI.
**PvRE**	Whole roots separated from fix+ nodules collected 21 days after inoculation.
**PvRI**	Whole roots separated from fix- nodules collected 21 days after inoculation.
**PvN5**	Pre-fixing (effective) nodules collected 5 days after inoculation.
**PvNE**	Effectively fixing nodules collected 21 days after inoculation.
**PvNI**	Ineffectively fixing nodules collected 21 days after inoculation.

https://www.zhaolab.org/PvGEA/
