# Supplementary material for: Genome-wide identification and comparative analysis of the Amino Acid Transporter (AAT) gene family and their roles during Phaseolus vulgaris symbioses
Source: Funct Integr Genomics. 2024 Mar 2;24(2):47. doi: 10.1007/s10142-024-01331-0 (PMC10908646; doi:10.1007/s10142-024-01331-0)
Supplement: Supplementary file 5 — Table S4 Primer sequences of Phaseolus vulgaris genes used to perform quantitative RT-PCR (PDF 29 kb) [file 10142_2024_1331_MOESM5_ESM.pdf]

**Table S4:** Primer sequences of *Phaseolus vulgaris* genes used to perform quantitative RT-PCR.

| ***Phaseolus vulgaris* gene** | **Oligonucleotide sequence (5' – 3')** | |
|---|---|---|
| *EIF4a* | **F** | *GGTGTCCACACTGTTGTTGG* |
| | **R** | *CGGCATTGTAGCAGAGAACA* |
| *IDE* | **F** | *GCAACCAACCTTTCATCAGC* |
| | **R** | *AGAAATGCCTCAACCCTTTG* |
| *CAT4* | **F** | *GGTTTGGGGTGTGGACAGGG* |
| | **R** | *AGGCACTTCCCCTTTTGCCC* |
| *PHS5* | **F** | *GAGGTTCAGCACGCCTTGGT* |
| | **R** | *ATGGTGGGCGACTTCCTCCT* |
| *ATLa5* | **F** | *TGCACCGCTTGCGAGCTTTA* |
| | **R** | *CTGTGGTGGCGTCTGGGAAG* |
| *AUX6* | **F** | *CGTCGCCGTCTATTGGGCTT* |
| | **R** | *ACCGTGCAAGTGCCCTCAAA* |
| *AAP14* | **F** | *TGGATGGACATGGCAGCAGG* |
| | **R** | *GCATGACAGCAGGACCAGCA* |
| *GAT2* | **F** | *AGTGATGGCACTGACTGCGG* |
| | **R** | *GCTGCTAAAACCGTGGCTGC* |
| *ProT3* | **F** | *GCAACCATCAGGCAGCCAGT* |
| | **R** | *GCCAGGGCACTCCCTTTGAT* |
| *LHT2* | **F** | *CCTGTGCAGCTGTGGTGCTA* |
| | **R** | *ATTGTGGCCCCGGAAAGCAA* |
